# How co-production helped to facilitate the development and implementation of the New Forest Parenting Programme in Denmark

**DOI:** 10.1192/j.eurpsy.2023.79

**Published:** 2023-07-19

**Authors:** A.-M. Lange

**Affiliations:** psychiatry, Aarhus university, Aarhus, Denmark

## Abstract

**Abstract:**

Evidence-based parent training is a widely recommended in the treatment of ADHD. However, most studies have not tested parent training with clinically referred children and practising clinicians in real-life clinical settings. In this presentation I will present findings from a randomised controlled trial funded by TrygFonden that evaluated the implementation of NFPP in hospital based child psychiatry clinics in the Denmark. The presentation will focus on the many implementation challenges that arose in translating a behavioural intervention from English into Danish. These include cultural adaptations and changes to mode of delivery that were required to ensure Danish parents would be able to engage with and access the intervention. Changes and adaptations were deeply informed by co-production with Danish parents and health care providers to ensure the successful development of NFPP in Denmark, appropriate evaluation, and widespread implementation within secondary care services in Denmark. The presentation will conclude with some preliminary findings from a second study funded by Trygfonden which is seeking to explore barriers to implementation of NFPP within Danish community based child and family services. Again, co-production has been invaluable in guiding the development of NFPP within Danish community care services. Identifying and overcoming barriers in implementation through co-production are essential parts of research needed to close the gap between intervention research and clinical practice

**Disclosure of Interest:**

None Declared

